# Cardiovascular responses to mild perinatal asphyxia in growth-restricted preterm lambs

**DOI:** 10.1152/ajpheart.00485.2023

**Published:** 2023-09-01

**Authors:** Matthew Oyang, Beth R. Piscopo, Valerie Zahra, Atul Malhotra, Amy E. Sutherland, Arvind Sehgal, Stuart B. Hooper, Suzanne L. Miller, Graeme R. Polglase, Beth J. Allison

**Affiliations:** ^1^The Ritchie Centre, Hudson Institute of Medical Research, Clayton, Victoria, Australia; ^2^Department of Obstetrics and Gynaecology, Monash University, Clayton, Victoria, Australia; ^3^Monash Newborn, Monash Children’s Hospital, Clayton, Victoria, Australia; ^4^Department of Paediatrics, Monash University, Clayton, Victoria, Australia

**Keywords:** cardiovascular physiology, fetal growth restriction, perinatal asphyxia, preterm birth, resuscitation

## Abstract

Growth-restricted neonates have worse outcomes after perinatal asphyxia, with more severe metabolic acidosis than appropriately grown neonates. The cardiovascular physiology associated with fetal growth restriction (FGR) may alter their response to asphyxia. However, research on asphyxia in FGR is limited. Here we compared cardiovascular hemodynamics in preterm FGR and control lambs during mild perinatal asphyxia. We induced FGR in one twin at 89 days gestation (term 148 days), while the other served as a control. At 126 days gestation, lambs were instrumented to allow arterial blood pressure and regional blood flow recording, and then mild perinatal asphyxia was induced by umbilical cord clamping, and resuscitation followed neonatal guidelines. FGR lambs maintained carotid blood flow (CBF) for 7 min, while control lambs rapidly decreased CBF (*P* < 0.05). Fewer growth-restricted lambs needed chest compressions for return of spontaneous circulation (ROSC) (17 vs. 83%, *P* = 0.02). The extent of blood pressure overshoot after ROSC was similar, but it took longer for MAP to return to baseline in FGR lambs (18.83 ± 0.00 vs. 47.67 ± 0.00 min, *P* = 0.003). Growth-restricted lambs had higher CBF after ROSC (*P* < 0.05) and displayed CBF overshoot, unlike control lambs (*P* < 0.03). In conclusion, preterm growth-restricted lambs show resilience during perinatal asphyxia based on prolonged CBF maintenance and reduced need for chest compressions during resuscitation. However, CBF overshoot after ROSC may increase the risk of cerebrovascular injury in FGR.

**NEW & NOTEWORTHY** Preterm growth-restricted lambs maintain carotid blood flow for longer than control lambs during asphyxia and have a lower requirement for chest compressions than control lambs during resuscitation. Preterm growth-restricted, but not control, lambs displayed an overshoot in carotid blood flow following return of spontaneous circulation.

## INTRODUCTION

Perinatal asphyxia is defined as a lack of blood flow to the fetus immediately before, during, or after birth. Although perinatal asphyxia is rare [incidence of 2/1,000–2/100 of all births in low and high-income countries, respectively (1)] it contributes to neonatal mortality and morbidity, accounting for 23% of neonatal deaths annually (1). Perinatal asphyxia causes cardiovascular complications including ischemic cardiac injury and ventricular failure (2). If clinical intervention following perinatal asphyxia is inadequate, ventricular failure occurs, causing cardiogenic shock and cardiac arrest (2). With timely resuscitation of the asphyxiated neonate, spontaneous circulation can be restored as myocardial function recovers (3), but return of spontaneous circulation (ROSC) may be accompanied by an overshoot in blood pressure, increasing the risk of cerebrovascular injury (4). The risk of cerebrovascular injury is likely to be higher in neonates with poor control of the cerebrovascular circulation such as those born premature or with fetal growth restriction (FGR) (5).

FGR affects 5–10% of pregnancies and is defined as estimated fetal weight < 10th centile for gestational age and sex, with abnormal fetal Doppler measurements, or fetal weight < 3rd centile on its own (6). FGR is a risk factor for perinatal asphyxia (7) and clinical evidence shows that growth-restricted neonates have a higher rate and severity of metabolic acidosis after perinatal asphyxia compared with appropriately grown neonates (8). Both preclinical and human data demonstrate that the cardiovascular physiology of FGR neonates is fundamentally different than appropriately grown neonates, including brain sparing in response to fetal hypoxia (9–11). A preclinical FGR study has also shown diminished autonomic, chemoreceptor, and baroreceptor response to an additional insult (12), thus increasing the risk of cardiovascular dysfunction during asphyxia. Few studies have specifically investigated cardiovascular hemodynamic response to asphyxia in FGR neonates.

This study compared cardiovascular hemodynamic responses to mild asphyxia in preterm FGR and control lambs. We hypothesized that FGR lambs would have impaired blood pressure regulation during asphyxia and altered cerebral blood flow response given that brain sparing is apparent in FGR offspring (7).

## METHODS

All experiments were approved by Hudson Institute Animal Ethics Committee (MMCA 2017/38) and conducted following National Health and Medical Research Council of Australia Code of Practice for the Care and Use of Animals for Scientific Purposes. Food and water were freely available, with food but not water withdrawn ∼18 h before surgery.

### Animal Preparation

Twin-bearing ewes (*n* = 6) were obtained from Monash Animal Research Platform and underwent aseptic surgery to carry out single umbilical artery ligation surgery at 89 days gestation (dGA, term 148dGA) for induction of early onset FGR in one twin, with the other twin acting as control (13). Our ovine model FGR induces early onset placental insufficiency and asymmetrical FGR consistent with the most common human form of FGR (13).

Ewes received betamethasone (11.4 mg; im) at 124dGA and 125dGA. At 126dGA, fetuses were partially removed from the uterus via cesarean section, and each lamb was intubated with a cuffed endotracheal tube, which was clamped to prevent spontaneous breathing. Ultrasonic flow probes (Transonic Systems) were placed around the femoral, left pulmonary, and left carotid artery for continuous recording of blood flows. Carotid blood flow (CBF) is a validated surrogate measure for cerebral blood flow in lambs (14). The left brachial artery, jugular vein, and femoral artery were catheterized. A near-infrared spectroscopy (NIRS) optode (Foresight, CAS Medical Systems) was placed on the lamb’s head to monitor regional cerebral oxygen saturation (crSo_2_). A transcutaneous arterial saturation probe (Massimo, Radical 7) was placed on the lamb’s tail to measure arterial saturation of oxygen ($Sa_{O_{2}}$) and heart rate (HR).

Before perinatal asphyxia was induced, baseline fetal recordings were obtained for 2 min. Asphyxia was induced by occluding the umbilical cord (UCO), withholding ventilation. While asphyxia was ongoing, the lamb was weighed and transferred to an infant warmer. UCO was continued until mean arterial pressure (MAP) declined to ∼25 mmHg. Volume-guaranteed ventilation was then commenced (Babylog 8000+; Draeger), marking the end of UCO. If HR remained below 60 beats/min despite 30 s of ventilation, chest compressions (90 compressions/min) were commenced, following international guidelines for neonatal resuscitation (15). Intravenous epinephrine (0.01–0.03 mg/kg, 1:10,000 epinephrine) was also administered if HR remained <60 beats/min despite 60 s of chest compressions and ventilation (15). Successful resuscitation (ROSC) was defined as HR > 100 beats/min and MAP > 30 mmHg.

Once successful resuscitation was achieved, lambs were sedated (10 mg/mL Alfaxan, Jurox), and ventilated for 1 h, with surfactant (100 mg/kg, Curosurf, Chiesi Farmaceutici) administration at 10 min after resuscitation. At the end of experimentation, lambs were euthanized (phenobarbitone; 100 mg/kg iv, Virbac). The FGR and control lamb in each twin pair were asphyxiated and resuscitated in a randomized order.

Arterial BP, blood flows, and crSo_2_ were digitally recorded in real time (1 kHz, PowerLab; ADInstruments). Regular arterial blood samples were analyzed (ABL700 Blood Gas Analyzer Radiometer). Baseline fetal data were obtained by averaging three randomly selected 10-s epochs before UCO, and the 10-s epoch immediately before UCO. During UCO, data were averaged over 5-s epochs every 10 s. The asphyxia time taken to reach 25 mmHg differed between animals but was at least 7 min in all animals. As such, we analyzed data for the first 7 min. After ROSC, data were averaged over 10-s epochs every 30 s for 1 h.

Femoral blood flow (FBF) and ventilation volume were adjusted for body weight, while CBF and pulmonary blood flow (PBF) were corrected for wet brain weight and lung weight, respectively.

Plasma levels of norepinephrine and epinephrine from FGR and control lambs were analyzed via ELISA following the manufacturer’s instructions (Labor Diagnostika Nord, LDN BA E-5200R and LDN BA E-5100R, respectively). Absorbance was determined using a plate reader (SpectraMax i3 Multi-Mode Platform, Molecular Devices) set at 450 nm.

An independent *t* test was used to assess statistical differences in fetal characteristics, interventions to achieve ROSC, and maximum mean MAP and ventilation parameters. Two-way repeated-measures ANOVA compared blood gas data. All other comparisons were compared using area under the curve (Prism v6; GraphPad Software) and subsequent *t* test. Data are presented as means ± SE unless otherwise stated. *P* value of <0.05 was considered significant.

## RESULTS

FGR lambs were hypoxic and weighed less than control lambs (Table 1). pH, $\text{P}{\text{a}}_{\text{C}{\text{O}}_{2}}$, lactate, and MAP were not different between groups during the baseline period but were altered with time in response to asphyxia. Blood gas parameters and MAP were not different between groups at the end of asphyxia (Table 1). The time taken for MAP to decline to 25 mmHg was not different between groups (Table 1).

**Table 1.** Fetal and end-asphyxia characteristics

	Control		FGR		
	Baseline	End asphyxia	Baseline	End asphyxia	*P* Value
Lambs, *n*	6		6		
Males, *n* (%)	3 (50)		4 (66)		0.60
Gestational age, days	126 ± 0		126 ± 0		
Body weight, kg	3.49 ± 0.20		2.46 ± 0.33*		0.02
pH	7.32 ± 0.01	6.95 ± 0.02#	7.36 ± 0.02	6.96 ± 0.02#	0.19
$\text{P}{\text{a}}_{\text{C}{\text{O}}_{2}}$, mmHg	22.0 ± 1.4	5.9 ± 0.0#	16.0 ± 1.6*	10.3 ± 1.2#	0.03
$\text{P}{\text{a}}_{\text{C}{\text{O}}_{2}}$, mmHg	59.6 ± 1.5	109.6 ± 4.3#	60.3 ± 2.5	115.0 ± 9.7#	0.82
$\text{S}{\text{a}}_{\text{C}{\text{O}}_{2}}$, %	57.6 ± 3.3	2.8 ± 1.2#	39.4 ± 5.5*	11.4 ± 5.8#	0.02
Lactate, mmol/L	4.9 ± 0.5	11.6 ± 0.9#	5.5 ± 0.6	12.4 ± 1.3#	0.46
Time taken to MAP of 25 mmHg, min	10.21 ± 1.13		12.25 ± 1.33		0.27
Chest compressions, %	83.3		16.67*		0.02
Epinephrine bolus, %	50		16.67		0.25

Values are means ± SE or percentages; *n*, number of lambs. FGR, fetal growth restriction; $\text{P}{\text{a}}_{{\text{O}}_{2}}$_,_ partial pressure of oxygen; $\text{P}{\text{a}}_{\text{C}{\text{O}}_{2}}$, partial pressure of carbon dioxide; $\text{S}{\text{a}}_{{\text{O}}_{2}}$, arterial saturation of oxygen; MAP, mean arterial pressure. *P < 0.05 statistical significance of FGR vs. control. Blood gas parameters determined with two-way repeated-measures ANOVA, with all other variables compared via Student’s *t* test. *#*Statistical significance of end asphyxia vs. baseline.

### Cardiovascular Hemodynamic Response to Asphyxia

**Figure 1.**
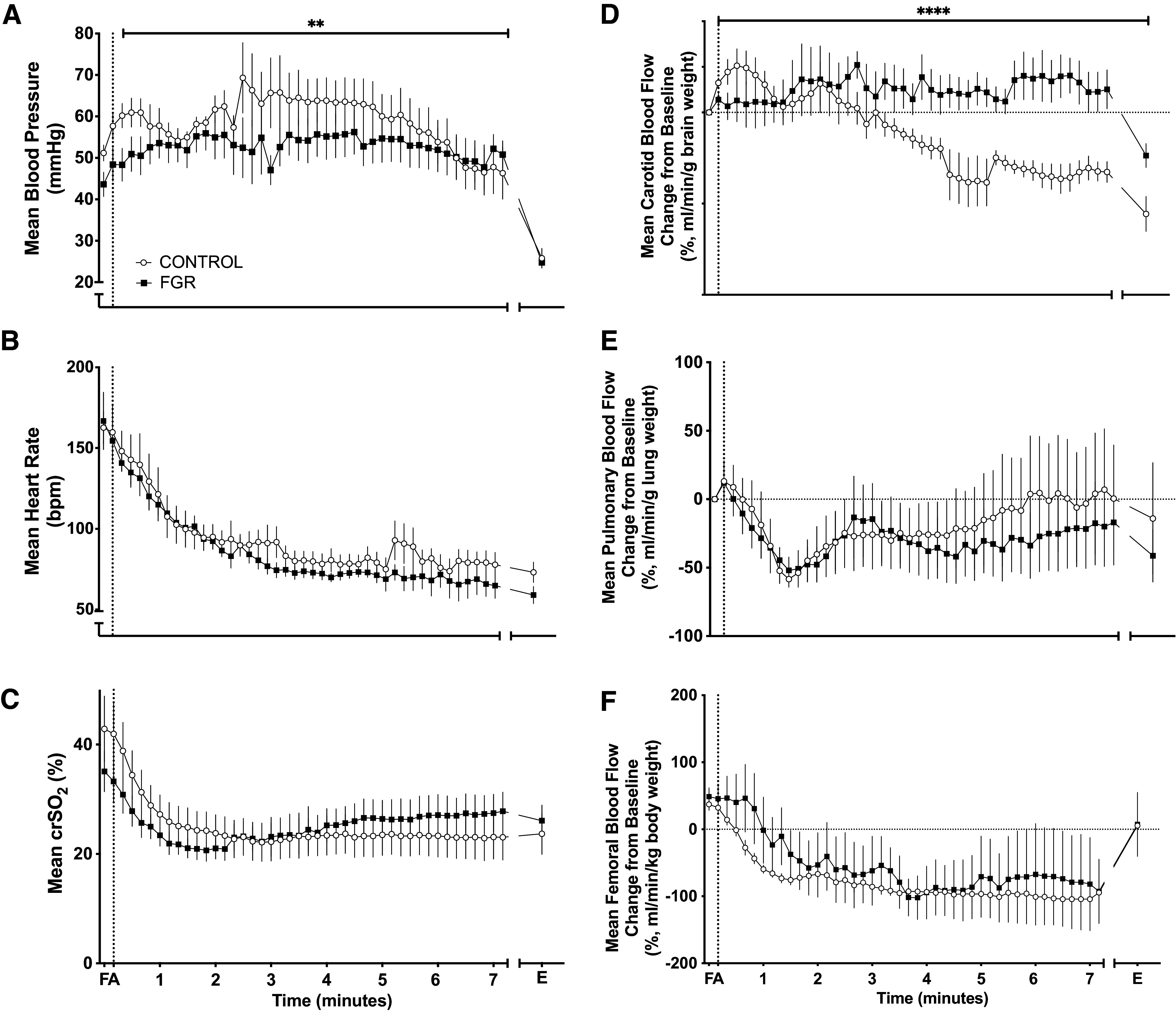
Hemodynamic response to mild asphyxia. Data are means ± SE for mean arterial pressure (*A*), mean heart rate (*B*), regional cerebral oxygen saturation (crSo_2_; *C*), and carotid (*D*), and pulmonary (*E*) and femoral (*F*) blood flow. Data are shown during the fetal period (*F*), at the start of asphyxia (A), and until the end of asphyxia (E) in control (○, *n* = 6) and fetal growth-restricted (FGR, ■, *n* = 6) lambs. **P < 0.01 and ****P < 0.0001, statistical significance in FGR vs. control via area under the curve with *t* test.

MAP was decreased in response to asphyxia, with control lambs having significantly higher MAP compared with FGR lambs (*P* = 0.0098, Fig. 1*A*). HR and crSo_2_ were significantly reduced in response to asphyxia but not different between FGR and control lambs (Fig. 1, *B* and *C*).

CBF progressively decreased in control lambs during asphyxia. In comparison, FGR lambs maintained stable CBF for the first 7 min of asphyxia, a response that was significantly different from control lambs (*P* < 0.001). Both control and FGR groups demonstrated a similar reduction in FBF and PBF in response to asphyxia.

### Resuscitation of Lambs

All lambs were successfully resuscitated. FGR lambs were less likely to require chest compressions during resuscitation compared with controls (16.67 vs. 83.3%, *P* = 0.02; Table 1). The proportion of lambs requiring epinephrine during resuscitation, although lower in the FGR group, was not different.

### Cardiovascular Hemodynamic Recovery from Asphyxia

**Figure 2.**
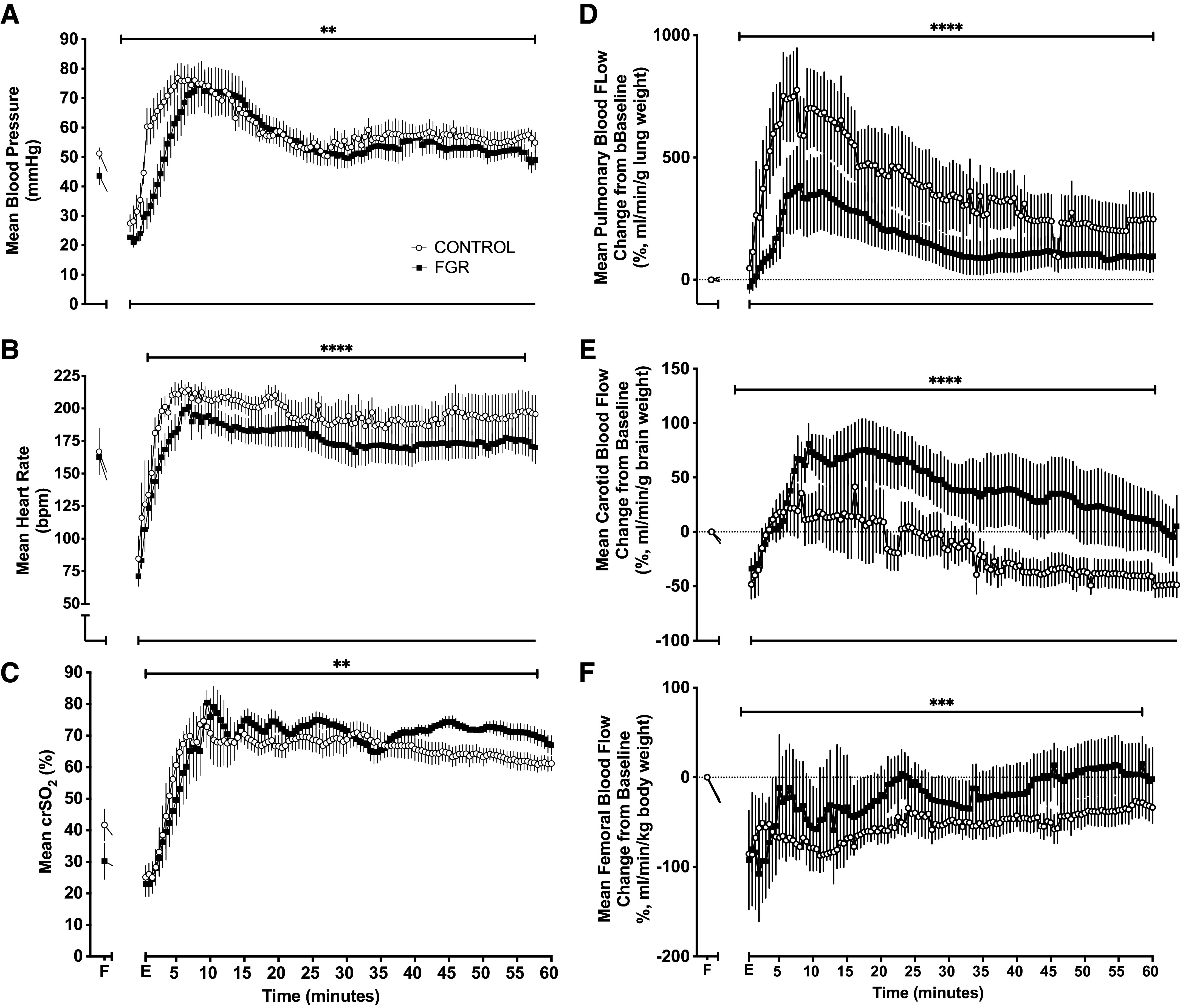
Hemodynamic recovery from mild asphyxia. Data are means ± SE for mean arterial pressure (MAP; *A*), heart rate (*B*), and carotid (*C*), pulmonary (*D*), and femoral (*E*) blood flow, and regional cerebral oxygen saturation (crSo_2_; *F*) measured during the fetal period (F) and until 60 min after the end of asphyxia (E) in control (○, *n* = 6) and fetal growth-restricted (FGR, ■, *n* = 6) lambs. **P < 0.01 and ***P < 0.001, and ****P < 0.0001, statistical significance in FGR vs. control via area under the curve with *t* test.

**Figure 3.**
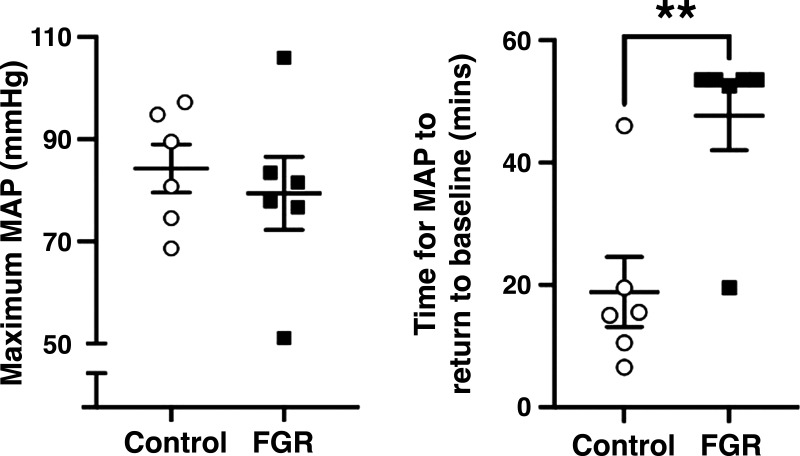
Properties of the blood pressure overshoot after asphyxia. Data are means ± SE for maximum mean arterial pressure (MAP; *A*) and time taken for MAP to return to baseline following the blood pressure overshoot (*B*) in control (○, *n* = 6) and fetal growth-restricted (FGR, ■, *n* = 6). **P < 0.01, statistical significance in FGR vs. control. Data were compared using an independent *t* test.

Following ROSC, MAP increased in FGR and control lambs resulting in a blood pressure overshoot in both groups (MAP significantly higher than baseline fetal MAP) (Fig. 2*A*). MAP increased more rapidly in control lambs compared with FGR lambs, resulting in an overall elevation of MAP during the recovery from asphyxia in control lambs (*P* < 0.001). The maximum MAP reached was not different between groups (Fig. 3). After ROSC MAP took ∼30 min longer to return to baseline in FGR compared with control lambs (18.8 ± 0.0 vs. 47.7 ± 0.0 min, *P* = 0.003; Fig. 3).

Following asphyxia, HR was increased above fetal levels in FGR and control lambs (*P* < 0.05). However, HR increased more rapidly in control lambs and was significantly higher compared with FGR lambs (*P* < 0.001) (Fig. 2*B*).

Cerebral tissue oxygen saturation obtained from the NIRS signal showed a rapid recovery following ROSC (Fig. 2*C*), with FGR lambs demonstrating a significant elevation of cerebral oxygenation compared with controls (*P* = 0.0029) for the hour after asphyxia (Fig. 2*E*).

PBF rapidly increased after ROSC, and control lambs showed a significantly greater response than FGR lambs (Fig. 2*D*). The CBF response was significantly different (*P* < 0.0001) between FGR and control lambs, wherein CBF showed a significant overshoot in FGR lambs compared with control lambs that were sustained for the hour following resuscitation (Fig. 3). FBF was significantly reduced in control lambs compared with FGR lambs (Fig. 2*F*).

### Plasma Norepinephrine and Epinephrine Concentration

Norepinephrine and epinephrine concentrations during the fetal period, end asphyxia, and 1-h post-ROSC were not different between groups.

## DISCUSSION

Growth-restricted neonates experience worse outcomes following perinatal asphyxia than their appropriately grown counterparts (8), but the reasons for this are unclear. Contrary to our hypothesis, our findings suggest that preterm growth-restricted lambs invoke a cardiovascular response during asphyxia that is potentially more protective than controls. Carotid blood flow was maintained for longer in FGR lambs during asphyxia, and they had a reduced requirement for chest compressions during resuscitation. These observations could be interpreted as the growth-restricted lambs, demonstrating a greater cardiovascular resilience to asphyxia. However, post-asphyxia, FGR lambs displayed a more sustained blood pressure and carotid blood flow overshoot, indicating a recovery from the perinatal asphyxia that is unique from that of control lambs. Sustained high blood pressure and cerebral blood flow may increase the risk of cerebrovascular damage in FGR infants (4).

Interestingly, we found that FGR lambs had a blunted initial response to asphyxia, not showing the typical transient increase in blood pressure as seen in control lambs. Progressive myocardial dysfunction causes blood pressure to fall during asphyxia (3, 16). FGR offspring already show evidence of myocardial alterations in both fetal and neonatal life (10, 17), albeit early in life these cardiac dysfunctions remain subclinical (17). Crispi et al. used echocardiographic assessments to demonstrate that cardiac dysfunction worsens with the severity of growth restriction (17). Our results support altered myocardial function in FGR offspring with subtle but significant differences in response to mild asphyxia.

The preservation of carotid blood flow during asphyxia in FGR lambs is likely due to chronic brain sparing in utero (5). The elevated carotid blood flow in FGR lambs corroborates previous findings in near-term growth-restricted sheep fetuses during acute hypoxia in utero (7). During asphyxia, the cerebral vasculature dilates and cerebral circulation becomes pressure-passive, such that fluctuations in systemic blood pressure will impact cerebral blood flow (16). In our study, the rate of blood pressure decline was similar between FGR and control lambs, and therefore, the maintenance of carotid blood flow in growth-restricted lambs was not mediated by blood pressure. Cerebral blood flow is also responsive to vascular tone within parenchymal arterioles (5). Myogenic, endothelial, neural, and metabolic mechanisms, and the partial pressure of carbon dioxide can all influence cerebral blood flow (18). We have previously shown neurovascular unit impairment (19) and impaired NO-induced vasodilation of cerebrovasculature in FGR (20). A preclinical study in FGR piglets showed increased oxygen extraction capacity compared with control piglets (21), with this line of study requiring further investigation.

An interesting finding within this study was the different distribution of blood into the systemic and pulmonary circulations in FGR and control lambs. In FGR lambs a lower PBF and higher systemic blood flows likely resulted from a higher pulmonary vascular resistance which reduced left-to-right shunting across the ductus arteriosus. As a result, the normal contribution of left ventricular output to PBF was reduced (22) and redirected toward the cerebral circulation and periphery arteries. Persistence of a high pulmonary vascular resistance increases the risk of pulmonary hypertension, known to be present in those born FGR (23).

Cardiac output is shown to be impaired in FGR (9–11), likely contributing to the delayed recovery from asphyxia. The blood pressure overshoot occurred more slowly and was sustained for longer in FGR lambs compared with control lambs, suggestive of slower cardiac function recovery. The extended arterial blood pressure overshoot in preterm growth-restricted lambs combined with increased cerebral blood flow in the post-asphyxia period, potentially places this population at a higher risk of cerebrovascular injury. Recovery of cardiac function is driven by catecholamines such as epinephrine and norepinephrine (24, 25) and α_1_-receptors on peripheral blood vessels. We found no difference in circulating catecholamine concentration; however, previous studies suggest that chronic exposure to hypoxia in utero results in hypoxemia-induced blunting of α_1_-receptors (12). The rebound tachycardia was also slower in FGR lambs following ROSC. Sympathetic activation of β_1_-receptors on the heart increases heart rate in the immediate period following ROSC (26). Yates et al. demonstrated altered β-receptor expression in the skeletal muscle of growth-restricted sheep fetuses (27); therefore, reduced responsiveness in heart rate may be due to impaired cardiac β_1_-receptor function or expression. However, it is also possible that the changes in cardiovascular hemodynamics demonstrated in this study have their origin in impaired brain stem control of cardiopulmonary function (28). Further studies will be needed to thoroughly investigate the mechanisms underpinning the altered hemodynamic responses observed here in FGR offspring.

### Limitations

Caution is needed when extrapolating our preclinical findings to human studies, especially given that the causes of FGR are heterogeneous, and here we are recapitulating the impact of placental insufficiency. Biomarkers of myocardial injury, such as troponin, would have provided complimentary assessment of the degree of asphyxia-induced myocardial damage, but we were unable to perform troponin assay because of an unavailability of a sheep-compatible enzyme-linked immunoassay kit at the time of this study. Furthermore, we acknowledge that the duration of this study was short, and examining the long-term impacts of asphyxia in FGR is critical.

### Conclusion

Our study compared cardiovascular responses to asphyxia in preterm growth-restricted and control lambs. We show that FGR lambs may have a more resilient cardiovascular response during asphyxia, indicated by a prolonged maintenance of carotid blood flow during asphyxia and reduced requirement for chest compressions during resuscitation. In the post-asphyxia period, we found that FGR lambs had a sustained blood pressure and carotid blood flow overshoot, which may place them at increased risk of cerebrovascular damage. Characterization of the mechanisms of vascular responsivity that are altered in FGR offspring, together with histological assessment of lamb brains, is required to determine whether the cardiovascular differences to asphyxia observed in FGR offspring are detrimental or beneficial.

## DATA AVAILABILITY

All data are described in the main text. Research data are available upon reasonable request.

## GRANTS

G.R.P., S.L.M, and B.J.A. are supported by National Health and Medical Research Council Investigator Grants 173731, 2016688, and 1175843, respectively.

## DISCLOSURES

No conflicts of interest, financial or otherwise, are declared by the authors.

## AUTHOR CONTRIBUTIONS

S.L.M., G.R.P., and B.J.A. conceived and designed research; M.O., B.R.P., V.Z., A.M., A.E.S., A.S., S.L.M., G.R.P., and B.J.A. performed experiments; M.O., B.R.P., A.M., S.L.M., and B.J.A. analyzed data; M.O., A.M., S.B.H., S.L.M., G.R.P., and B.J.A. interpreted results of experiments; M.O. and B.J.A. prepared figures; M.O., B.R.P., V.Z., A.E.S., A.S., S.L.M., G.R.P., and B.J.A. drafted manuscript; M.O., B.R.P., V.Z., A.M., A.E.S., A.S., S.B.H., S.L.M., G.R.P., and B.J.A. edited and revised manuscript; M.O., B.R.P., V.Z., A.M., A.E.S., A.S., S.B.H., S.L.M., G.R.P., and B.J.A. approved final version of manuscript.
